# Announcement of Lectures in the Cleveland Medical College for the Session 1848-9

**Published:** 1848-10

**Authors:** 


*Announcement of Lectures in the Cleveland Medical College for the Session* 1848–9.

We quote from this circular the following paragraph:—

"The members of the Board of Instruction filling practical departments, have not only possessed advantages for making themselves familiar with the progress of medical Science and literature, but also, by an extended practice of their profession during a long series of years, have been able to mature practical views from their own large experience. Medical science and experience conjoined, will be very much divested of mere theories, and when at the command of those long accustomed to instruct by Lectures, will not fail to make a direct and permanent impressions on the minds of those desirous of becoming good Physicians.

The expression, "practical departments," which, in itself, is not very explicit, will be understood, by reference to the names of the Faculty, to apply to *General Pathology, Midwifery, and diseases of children*, and of *Physical diagnosis, and the theory and practice of Physic.* The reader will perhaps surmise that these chairs in some other Institutions are filled by persons less advanced in years than the incumbents of the Cleveland College.

